# PCSK9 Inhibitors and Anthracyclines: The Future of Cardioprotection in Cardio-Oncology

**DOI:** 10.3390/hearts5030027

**Published:** 2024-09-03

**Authors:** Matthew L. Repp, Mark D. Edwards, Christopher S. Burch, Amith Rao, Ikeotunye Royal Chinyere

**Affiliations:** 1 Department of Medicine, University of Colorado, Aurora, CO 80045, USA; 2 Department of Medicine, University of Michigan, Ann Arbor, MI 48109, USA; 3 Arizona College of Osteopathic Medicine, Midwestern University, Glendale, AZ 85308, USA; 4 Department of Medicine, Banner University Medicine, Tucson, AZ 85724, USA; 5 Sarver Heart Center, University of Arizona, 1501 North Campbell Avenue, Room 6154, Tucson, AZ 85724, USA

**Keywords:** chemotherapy, cardiology, heart failure, cancer, prophylaxis, pleiotropic

## Abstract

The field of cardio-oncology is an expanding frontier within cardiovascular medicine, and the need for evidence-based guidelines is apparent. One of the emerging focuses within cardio-oncology is the concomitant use of medications for cardioprotection in the setting of chemotherapy regimens that have known cardiovascular toxicity. While clinical trials focusing on cardioprotection during chemotherapy are sparse, an inaugural trial exploring the prophylactic potential of Sodium-Glucose Cotransporter-2 inhibitors (SGLT2is) for anthracycline (ANT)-induced cardiotoxicity has recently commenced. Proprotein convertase subtilisin/kexin type 9 (PCSK9) inhibitors, though less studied in this oncology demographic, have exhibited promise in preclinical studies for conferring cardiac protection during non-ischemic toxic insults. While primarily used to reduce low-density lipoprotein, PCSK9 inhibitors exhibit pleiotropic effects, including the attenuation of inflammation, reactive oxygen species, and endothelial dysfunction. In ANT-induced cardiotoxicity, these same processes are accelerated, resulting in premature termination of treatment, chronic cardiovascular sequelae, heart failure, and/or death. This review serves a dual purpose: firstly, to provide a concise overview of the mechanisms implicated in ANT-induced cardiotoxicity, and, finally, to summarize the existing preclinical data supporting the theoretical possibility of the cardioprotective effects of PCSK9 inhibition in ANT-induced cardiotoxicity.

## 1. Introduction

Anthracyclines (ANT), including doxorubicin (DOX), daunorubicin, epirubicin, and idarubicin, are chemotherapeutic drugs that have been widely effective in the treatment of various hematologic and solid malignancies since their introduction into clinical oncology in the 1960s. In the early stages of their therapeutic utilization, ANT-induced cardiotoxicity became a well-documented adverse effect that occurs in a single- and cumulative-dose-dependent manner [1].

Even after several decades of research, dexrazoxane (DZR) remains the only Food and Drug Administration (FDA)-approved cardioprotective medication for ANT-induced cardiac toxicity. It is exclusively approved for a narrow demographic, specifically adults with advanced metastatic breast cancer with ≥300 milligrams per square meter (mg/m^2^) of lifetime doxorubicin exposure, yet requiring additional ANT maintenance therapy [2]. Current cardio-oncology guidelines and expert recommendations regarding prophylactic interventions lack compelling evidence from properly powered clinical trials. Notably, conventional heart failure medications, including angiotensin-converting enzyme inhibitors, beta-blockers, angiotensin receptor blockers, and statins, demonstrate negative or conflicting results [3,4].

In patients both with and without type two diabetes mellitus, Sodium-Glucose Cotrans porter-2 inhibitors (SGLT2is) exert numerous cardiovascular benefits and have been shown to reduce the incidence of cardiovascular death and heart failure exacerbations leading to hospitalizations [5]. The Empagliflozin in the Prevention of Cardiotoxicity in Cancer Patients Undergoing Chemotherapy Based on Anthracyclines (EMPACT; NCT05271162) [6] is an inaugural randomized, multi-center, placebo-controlled, double-blind clinical study investigating the use of prophylactic empagliflozin for ANT-induced cardiotoxicity. Though less studied in this oncology demographic, proprotein convertase subtilisin/kexin type 9 (PCSK9) inhibitors have produced promising results in preclinical studies for conferring cardiac protection in the setting of non-ischemic toxic insults.

PCSK9 is a proprotein convertase that binds to, and subsequently degrades low-density lipoprotein (LDL) receptors on the surface of hepatocytes. Evolocumab and alirocumab are monoclonal antibodies that inhibit the normal function of PCSK9. This inhibition increases the presence of LDL receptors in the liver, allowing for increased uptake of LDL from the systemic and portal circulation, making it an effective drug for treating hypercholesteremia. While primarily used for its LDL-reducing capacities, PCSK9 inhibitors, much like SGLT2is, exhibit pleiotropic effects, including the attenuation of inflammation, reactive oxygen species (ROS), and endothelial dysfunction.

In ANT-induced cardiotoxicity, these same processes are accelerated, resulting in premature termination of treatment, chronic cardiovascular sequelae, heart failure, and/or death. Recent evidence [7–9] suggests that PCSK9 inhibitors may play a role in prophylactic cardioprotection in patients undergoing ANT-based chemotherapies. However, presently no clinical trials involving PCSK9 inhibitors are being conducted for oncology patients that have been exposed to anthracyclines. This review aims to succinctly describe the mechanisms involved in ANT-induced cardiotoxicity, followed by a summary of the existing preclinical data on the cardioprotective effects of PCSK9 inhibition.

## 2. Mechanisms of ANT-Induced Cardiotoxicity

**Figure 1.**
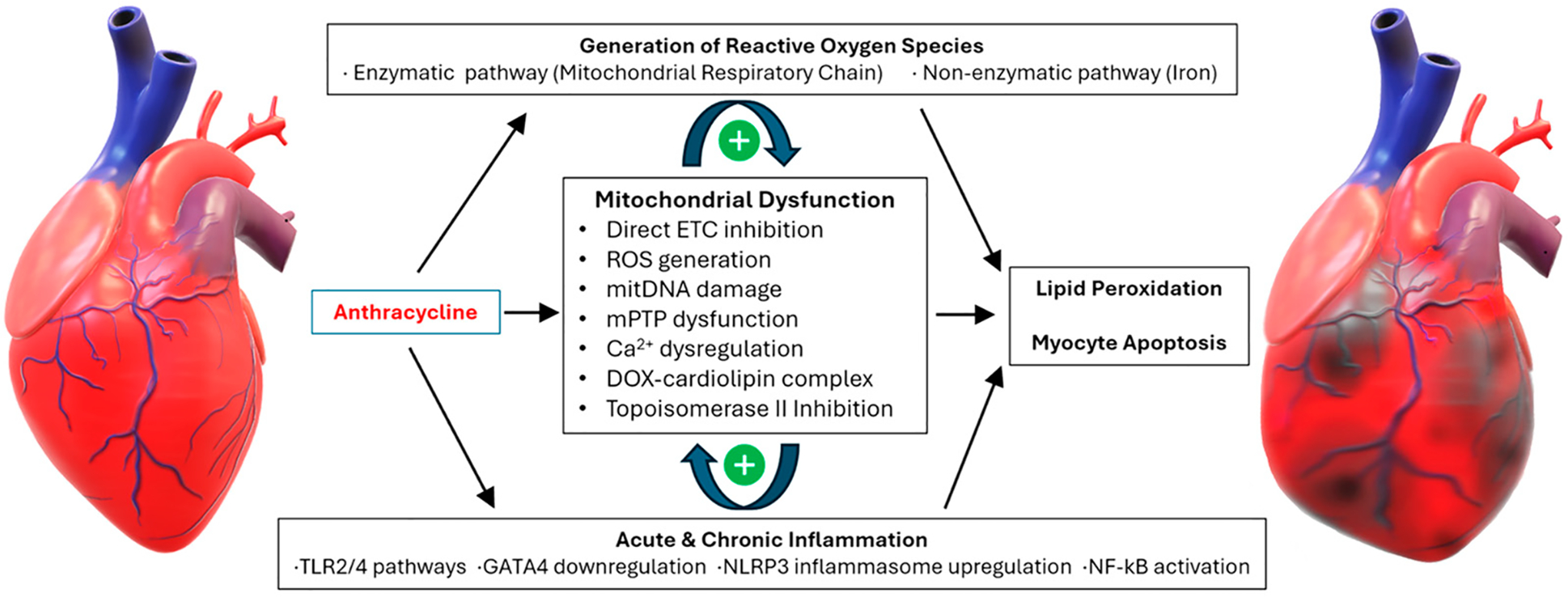
Mechanisms of Anthracycline-induced Cardiotoxicity. Anthracyclines are known to induce mitochondrial dysfunction directly via calcium dysregulation in addition to other toxic mechanisms. Anthracyclines are also capable of generating increased reactive oxygen species and non-specific, innate-mediated inflammation. These mechanisms consolidate into increased lipid peroxidation, affecting cardiomyocytes and microvasculature, resulting in chemotherapy-induced cardiomyopathy. Abbreviations: Ca^2+^—calcium; DOX—doxorubicin; ETC—electron transport chain; mitDNA—mitochondrial DNA; mPTP—mitochondrial permeability transition pore; NLRP3—nucleotide oligomerization domain-like receptor protein inflammasome; NF-κB—nuclear Factor kappa-light-chain-enhancer of activated B cells; ROS—reactive oxygen species; TLR—toll-like receptor. Illustrations utilized with permission from Microsoft (Word, Version 2408 Build 16.0.17928.20114) for academic/educational purposes.

ANT induce their cardiotoxic effects through several distinct yet related mechanisms (Figure 1), namely the production of ROS, mitochondrial dysfunction, binding to deoxyribonucleic acid (DNA) and topoisomerase II (Top2), as well as inducing innate inflammation.

### 2.1. Reactive Oxygen Species

The primary cardiotoxic mechanism associated with DOX is the production of free radicals and ROS. ANT causes free radical production through two distinct redox cycling pathways: an enzymatic pathway involving reduced nicotinamide adenine dinucleotide phosphate (NADPH)-Cytochrome P-450 reductase within the mitochondrial respiratory chain, and a non-enzymatic pathway dependent on iron and the Fenton reaction [10]. These radicals surpass the heart’s antioxidant defense systems, resulting in cardiomyocyte damage. Cardiomyocytes operate predominately via oxidative metabolism, necessitating a higher density of mitochondria compared to other cell types. DOX-induced free radical generation induces mitochondrial dysfunction, which leads to a cycle of organelle damage, oxidative stress, and ultimately myocyte apoptosis [1].

### 2.2. ANT-Induced Mitochondrial Dysfunction

#### 2.2.1. ANT-Induced Oxidative Stress

DOX is a cationic complex molecule with a high affinity for forming a DOX-cardiolipin complex. Cardiolipin is an anionic phospholipid within the inner mitochondrial membrane, and the DOX-cardiolipin complex impairs mitochondrial functioning [11,12]. In addition, DOX has a propensity to accumulate within mitochondria and nuclei, resulting in mitochondrial toxicity through various mechanisms. DOX can directly affect the respiratory chain by inhibiting complex I and other enzymes necessary for oxidative phosphorylation [13]. The overwhelming production of ROS, in part due to disruption of the respiratory chain, further propagates mitochondrial damage and results in lipid peroxidation. Cardiolipin peroxidation from ROS stimulates apoptotic pathways and subsequent myocyte cell death [14,15].

#### 2.2.2. Mitochondrial Permeability Transition Pore

The mitochondrial permeability transition pore (mPTP) is localized to the inner mitochondrial membrane and is regulated by intramitochondrial calcium (Ca^2+^) accumulation and redox imbalance [16]. Studies have shown that in conditions of mitochondrial calcium overload, the mPTP opens and permits the entrance of small molecular weight cofactors and cations, causing disruption of metabolic gradients between the mitochondria and cytosol, leading to mitochondrial swelling and eventual rupture of the outer membrane. In the context of myocardial tissue damage from DOX, intramitochondrial Ca^2+^ increases concomitantly with mitochondrial dysfunction, causing the mPTP to release pro-apoptotic proteins that activate apoptotic and necrotic cell death pathways [17]. The amphipathic properties of DOX facilitate its easy passage across organelle membranes, resulting in substantial accumulation in the mitochondria at concentrations exceeding 100 times that of normal plasma levels [18]. DOX significantly disrupts oxidative phosphorylation, as described above, and this stress can trigger the opening of the mPTP and subsequently lead to cell apoptosis or necrosis.

### 2.3. Doxorubicin-DNA Complexes and Topoisomerase II Inhibition

DOX plays a crucial role in inhibiting Top2, which is vital for DNA replication and chromosome structure maintenance. DOX targets two distinct isozymes of Top2: Top2a and Top2b. Top2a is only expressed in proliferating and tumor cells, and plays a role in DNA replication, chromosome degradation, condensation, and segregation. Top2b, on the other hand, is expressed in all healthy cells, including non-mitotic cells and contributes to transcriptional regulation [19]. DOX targets the Top2a isozyme creating a Top2-DOX-DNA cleavage complex that leads to lethal double-strand DNA breaks, initiating cardiac cell death cascades. This cleavage complex also stimulates ROS production, as well as dysfunction of mitochondrial biogenesis, further promoting cardiac cellular disruption [20]. The mechanism of dexrazoxane with respect to cardioprotection from DOX is hypothesized to reside within iron chelation, as well as depletion of Top2a and Top2b [21], the latter mitigating DNA damage.

### 2.4. ANT-Induced Inflammation

The culmination of DOX-induced ROS formation, mitochondrial dysfunction, and myocyte DNA damage manifests as innate-mediated inflammation of the myocardium.

#### 2.4.1. Toll-like Receptors and NF- κB

When ANT damages cardiac myocytes, a continuous release of damage-associated molecular patterns such as the high-mobility group protein B1 [22] ensues. This protein is capable of binding to toll-like receptors (TLRs) on myeloid cells, resulting in the recruitment of the adaptor protein molecule myeloid differentiation primary response 88 (MyD88) to the receptor-ligand complex [23]. Through signal transduction, the nuclear factor kappalight-chain-enhancer of activated B cells (NF-κB) induces pro-inflammatory cytokines. TLR2 and TLR4 are MyD88-mediated signaling pathways implicated in the pathogenesis of DOX-induced cardiomyopathy in mice [24,25], myocardial inflammation, ischemia-reperfusion (I/R) injury, and heart failure [23]. The transcription factor GATA-4 regulates cardiac myocyte gene expression as well as apoptosis and cell survival [26]. DOX-mediated activation of the TLR4 receptor down-regulates GATA4 activity and subsequently induces myocyte apoptosis [25,26].

#### 2.4.2. NOD-like Receptor Protein Inflammasome

The nucleotide oligomerization domain (NOD)-like receptor protein (NLRP3) inflammasome has been aggressively studied due to its implication in numerous inflammatory-related disorders [27]. The inflammasome is activated by harmful stimuli such as pathogens and/or cellular stress, which causes the activation of caspase-1 and which results in the up-regulation of proinflammatory cytokines Interleukin (IL)-1β/IL-18 [27,28]. These cytokines activate cardiomyocyte apoptosis pathways, resulting in adverse cardiac remodeling and impaired contractility. DOX has been shown to enhance the activation of the NLRP3 inflammasome, with previous studies demonstrating dose-dependent increases in IL-1β levels in patients treated with ANT regimens [29].

### 2.5. Emerging Research and Clinical Perspectives

DZR was approved by the FDA in 1997 for its cardioprotective effects against ANT-induced cardiotoxicity, based on the findings of two multicenter, double-blind studies (Multicenter Trials 088001 and 088006). It has long been hypothesized that DZR mitigates cardiotoxicity by chelating iron, thereby reducing ROS production through the Fenton reaction. Recent evidence also indicates that DZR depletes Top2a and Top2b isoforms, further helping to mitigate cardiomyocyte DNA damage. In 534 patients with advanced breast cancer, DOX with DZR showed a significant reduction in left ventricular ejection fraction (LVEF) decline from baseline, as well as new onset congestive heart failure during treatment when compared to DOX and a placebo treatment arm [30]. Clinical trials involving statins, angiotensin II receptor antagonists, beta blockers [31], and angiotensin-converting enzyme inhibitors [32] have shown no significant differences between treatment arms at worst, and only transient protective effects with conflicting results at best, with no reduction in heart failure incidence. Clinically, this positions DZR as the only proven cardioprotective medication for patients receiving ANT-based treatments. It is specifically recommended for the prevention of toxicity in a select group of adults classified as high and very high risk, particularly those with exposure levels of ≥300 mg/m^2^ [33]. Zheng and Zhan present a compelling clinical perspective, asserting that DZR has the strongest evidence for cardioprotection and should be considered for appropriate patients. They recommend continuing neurohormonal antagonists for other cardiovascular indications, and suggest initiating statins in higher-risk patients, given some benefits observed in clinical trials [31].

The various mechanisms underlying ANT-induced cardiotoxicity highlight the necessity of targeting multiple pathways while addressing their self-perpetuating nature and how they propagate one another. These mechanisms likely explain why DZR can attenuate cardiotoxicity but not completely prevent it. Similar to guideline-directed medical therapy, polypharmacy modulates pathologic pathways, and emerging research and perspectives are suggesting polypharmacy assists with cardioprotection synergistically. The eagerly anticipated results of the inaugural EMPACT trial will shed light on how the known anti-inflammatory and metabolic effects, as well as the cardioprotective properties of SGLT2is, may benefit patients receiving ANT treatments.

## 3. PCSK9 Inhibitors in ANT-Induced Cardiotoxicity

### 3.1. Protection from Oxidative Stress

For the past five decades, the leading hypothesis explaining ANT-induced cardiotoxicity has focused on redox cycling pathways and the iron-mediated formation of cardiac oxidative stress [1,34]. Despite achieving some success in cellular studies and acute animal models [35], the mitigation of ANT-induced cardiotoxicity using established antioxidants has proven underwhelming with regard to preventing cardiac damage in both chronic-exposure animal models and oncology patients [36]. Preclinical leads with strong promise to mitigate ANT-induced cardiotoxicity have yet to translate to effective clinical medicine. Clinical trials investigating the protective and reversal effects of antioxidants on cardiotoxicity have examined various substances, including *N*-acetylcysteine [37,38], oral glutathione [39], vitamin E [40], flavonoids [41] (Table 1), as well as candesartan and carvedilol [42–44]. Results have varied, with some showing negative outcomes or conflicting results, particularly regarding the effectiveness of carvedilol.

Exhaustive literature searches reveal no publicly available studies examining the antioxidant potential of PCSK9 inhibitors in the context of ANT-induced cardiotoxicity. However, the non-LDL receptor-based mechanisms of PCSK9 antagonism likely overlap with the known pathways that mediate ANT-induced cardiotoxicity. This suggests a potential prophylactic use of PCSK9 inhibitors in preventing chemotherapy-mediated cardiotoxicity.

PCSK9′s non-LDL receptor-mediated pathways have yet to be elucidated in detail regarding the mechanisms underlying oxidative stress and inflammation, yet their associations are not lacking evidence. Studies have implicated PCSK9s in the generation of, and cross-talk between, oxidative stress and chronic inflammation, especially concerning atherosclerosis formation [45,46]. Ding et al. showed compelling evidence that there is bidirectional crosstalk between PCSK9 and ROS generation. Under low shear stress conditions, vascular smooth muscle and endothelial cells increased PCSK9 expression as well as the generation of ROS [47].

Interestingly, specific NADPH oxidase inhibitors (diphenylene-iodonium chloride and apocynin) caused a significant reduction in PCSK9 expression. Conversely, PCSK9 knockdown significantly decreased the generation of ROS while PCSK9 overexpression increased ROS generation in a dose-dependent manner [47]. Another study found that prophylactic administration of evolocumab to human umbilical vein endothelial cells exposed to H_2_O_2_ had antioxidant and cytoprotective effects, evidenced by significantly decreased hydroperoxide concentrations, decreased lipid peroxidation caused by ROS, and increased ferric-reducing antioxidant power [48]. Inhibition of PCSK9 with alirocumab in rats with alcohol-mediated liver damage attenuated 4-hydroxy-2-nonenal (a byproduct of lipid peroxidation leading to cellular apoptosis of hepatocytes), showing antioxidant certainty of these drugs. Myeloperoxidase-positive neutrophils that generate ROS were also significantly decreased in the liver when treated with alirocumab, compared to control groups [49].

### 3.2. Protection from Mitochondrial Dysfunction

A detrimental positive-feedback loop ensues as ANT induces mitochondrial damage, while compromised mitochondria produce ROS, contributing to chronic cardiotoxicity. Fission-fusion dynamics maintain mitochondrial health during physiologic and environmental stress [50]. Myocytes are heavily reliant on oxidative metabolism for energy generation, with mitochondria comprising approximately 36% of cardiac cellular volume [51]. The heart’s high mitochondrial density and reliance on proper mitochondrial function render it particularly susceptible to damage when ANT causes widespread mitochondrial dysfunction [1]. Data support the view that inhibiting PCSK9 offers the promise of mitigating mitochondrial malfunction, which may be extrapolated to prophylactically alleviate ANT-induced cardiotoxicity.

PCSK9 overexpression has been linked to mitochondrial dysfunction. Elevated PCSK9 levels in vascular smooth muscle have been associated with mitochondrial apoptosis, as well as mitochondrial fission and subsequent mitochondrial dysfunction [52]. There is a bidirectional cross-talk between PCSK9 and mitochondrial DNA (mitDNA) damage, wherein inhibiting PCSK9 reduces mitDNA damage, while inducing mitDNA damage increases PCSK9 levels, ultimately causing cellular apoptosis [53]. In a murine model, Li et al. demonstrated that PCSK9 inhibition alleviated the deleterious effects of oxidized (ox)-LDL by decreasing dynamin-related protein 1-mediated mitochondrial fission, ROS, and cardiomyocyte apoptosis. Lectin-like oxidized low-density lipoprotein receptor-1 (LOX-1) knockdown also decreased ox-LDL/LOX-1 myocardial damage and PCSK9 expression, which is overexpressed in the presence of ox-LDL [54]. PCSK9 also regulates pyroptosis through mitDNA damage and activation of the NLRP3 inflammasomes [55]. Interestingly, PCSK9 inhibits ubiquinol-cytochrome c reductase core protein 1 protein expression (mitochondrial respiratory chain complex III subunit necessary for proper mitochondrial function), resulting in mitochondrial dysfunction and increased oxidative stress [56].

I/R injury shares mechanisms similar to those driving ANT-induced cardiotoxicity. In Sprague-Dawley rats (10–12 weeks old) undergoing cardiac I/R injury, elevated PCSK9 levels triggered mitophagy through the Bcl-2/adenovirus E1B 19-kDa interacting protein pathway in cardiomyocytes [57]. This overexpression also induced autophagy in cardiomyocytes, exacerbating the progression of myocardial infarction. Additionally, heightened PCSK9 expression worsened reperfusion injury, leading to increased myocardial scarring and cardiac dysfunction. However, prophylactic administration of evolocumab resulted in decreased mitophagy and autophagy, ultimately alleviating both I/R injury and cardiac dysfunction [57].

### 3.3. Protection from Inflammation

Inflammatory insults to cardiac tissue and ineffective reparative responses set the stage for chronic inflammation that culminates in progressive cardiovascular disease [58]. Both animal and clinical studies indicate that PCSK9 levels correlate with increased vascular and systemic inflammation, which can be mitigated by PCSK9 deficiency or inhibition [59]. Although the mechanisms have not been fully elucidated, the Canakinumab Antiinflammatory Thrombosis Outcome Study (CANTOS; NCT01327846) trial demonstrated that chronic inflammation of the myocardium plays a significant role in heart failure pathophysiology. When compared to placebo, patients with previous myocardial infarction and who were given canakinumab, a monoclonal antibody directed against IL-1β, had a significantly lower incidence of recurrent cardiovascular events, as well as significantly lower high sensitivity C-reactive protein [60].

Studies have demonstrated that PCSK9 stimulates the NLRP3 inflammasome pathway [55,61,62] and that NLRP3 activation increases PCSK9 secretion, predominantly through IL-1β/IL-18 cytokine signaling [63]. Overexpression of PCSK9 is associated with the transformation of cardiac fibroblasts into myofibroblasts and cardiac fibrosis [64], as well as vascular and systemic inflammation [59]. Interestingly, the cardiovascular outcomes in the CANTOS trial through blocking IL-1β were similar to outcomes seen in landmark studies that investigated clinical outcomes with PCSK9 inhibition [65,66].

DOX upregulates the IL-1 signaling pathway and increases local and systemic inflammation, which are responsible for its dose-dependent adverse effects [67]. A study involving a murine model was conducted, which utilized a recombinant human IL-1 receptor antagonist, and it showed significant improvements in LVEF, decreased markers of cardiotoxicity (troponin I and malondialdehyde), and decreased myofibrillar loss in mice injected with DOX [68]. Another study was conducted utilizing a direct NLRP3 inflammasome inhibitor (JC121), which showed similar efficacy in limiting myocardial fibrosis and left ventricular systolic dysfunction in DOX-injected mice [69]. This demonstrates the pivotal role that the NLRP3 inflammasome plays in the cardiotoxic effects of anthracycline regimens. Given their activation by PCSK9, it also offers insight into PCSK9 inhibition as a potential therapy that can mitigate these inflammatory effects.

Quagliariello et al. [7] exposed a human fetal cardiomyocyte cell line to DOX alone or in sequence with trastuzumab, followed by ± co-incubation with evolocumab. They found that evolocumab enhanced cell viability by 35–43% (*p* < 0.05) in both DOX ± trastuzumab groups compared to the groups that did not receive evolocumab. Pro-inflammatory studies showed that evolocumab mitigated the cardiotoxicity of DOX alone and with trastuzumab. A similar study found that human fetal cardiomyocytes exposed to DOX, trastuzumab, and nivolumab ± evolocumab co-incubation improved cell viability from 38 to 59% (*p* < 0.05), and downregulated cardiotoxic pro-inflammatory pathways [8].

Another study proposed that the cardioprotective effects of SGLT2is may be due, in part, to PCSK9 pathways. In mice treated with DOX and dapagliflozin for 10 days, there was a statistically significant reduction in PCSK9 levels, IL-1β, and CRP levels compared to the DOX group alone, suggesting that there may be SGLT2/PCSK9 cross-talk pathways [9]. The dapagliflozin + DOX-treated group showed decreased myocardial and hepatic NLRP3 inflammasome expression compared to the DOX group alone, which could also be attributed to deceased PCSK9 expression.

## 4. Discussion

This review aimed to: (1) describe the mechanisms involved in ANT-induced cardiotoxicity, and (2) summarize the existing preclinical data on the potential cardioprotective effects of PCSK9 inhibition for ANT-induced cardiotoxicity. Cardiotoxicity induced by ANT, illustrated primarily via DOX exposure, manifests primarily via induction of non-specific inflammation via the innate immune system [11–13], increased generation of free radicals and subsequent ROS, mitochondrial dysfunction via disruptions in aerobic respiration, cation handling, and mitochondrial DNA damage [16,25,26]. These processes ultimately lead to the induction of cardiomyocyte apoptosis, contributing to the clinical manifestation of heart failure. Additionally, they induce lipid peroxidation that accelerates the atherosclerosis burden and increases the risk of ischemic cardiovascular disease.

**Figure 2.**
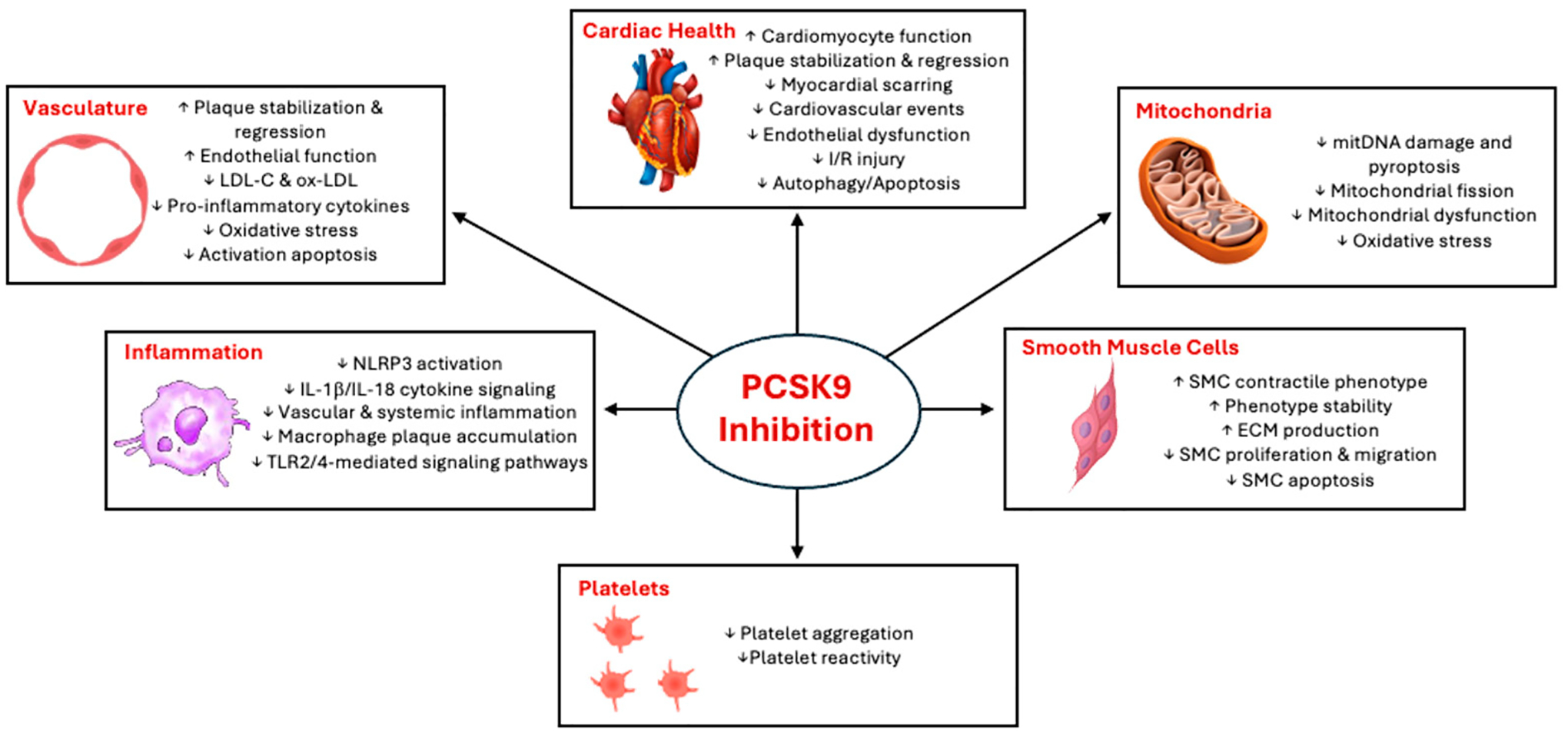
Pleiotropic effect of PCSK9 antagonism. PCSK9 inhibitors primarily reduce LDL-C but also exhibit several pleiotropic effects. Most notably, they stabilize atherosclerotic plaques, decrease pro-inflammatory cytokines, improve cardiomyocyte function, reduce mitochondrial dysfunction, and enhance smooth muscle cell function. These multiple pleiotropic effects may be particularly cardioprotective against anthracycline-induced cardiotoxicity. Abbreviations: ECM—extracellular matrix; IL—interleukin; I/R—ischemia-reperfusion; LDL-C—low-density lipoprotein cholesterol; mitDNA—mitochondrial DNA; NLRP3—nucleotide oligomerization domain-like receptor protein inflammasome; Ox—oxidized; PCSK9—proprotein convertase subtilisin/kexin type 9; SMC—smooth muscle cell; TLR—toll-like receptor. Illustrations utilized with permission from Microsoft (Word, Version 2408 Build 16.0.17928.20114) for academic/educational purposes.

Although limited, preclinical and clinical data demonstrate that PCSK9 inhibition holds theoretical promise as a potential avenue for treatment of anthracycline-induced cardiotoxicity (Figure 2). In both animal models and human studies, the quantity of PCSK9 correlated positively with the degree of granulocyte-mediated and pathologic ROS [47], inflammation [59], and mitDNA damage [53]. Inhibition of PCSK9 or induced deficiency of PCSK9 has been found to mitigate the aforementioned consequences, and has been associated with improved aerobic cellular respiration [56] as well as cytoprotective effects. These findings, although yet to be formally evaluated in a properly run clinical study, are drawing the attention of physicians and patients alike. With the average life expectancy of oncology patients continuing to rise, novel challenges are emerging for cancer survivors who require evidence-based interventions. It is important to note that no known contraindications exist regarding the co-administration of any common chemotherapy agent and PCSK9 inhibitors.

Current gaps in preclinical and clinical investigations that will be necessary prior to the therapeutic use of PCSK9 inhibitors for cancer patients are: (1) the validation of the mechanism of action and/or theurapetic index in an animal model of ANT-induced cardiotoxicity with strong clinical correlation and similar propensity to mitochondrial dysfunction, (2) an improved understanding of the short-, medium-, and long-term side-effects or unintended consequences of PCSK9 inhibition in patients with acquired cardiovascular disease, and (3) a preliminary proof-of-concept clinical cohort trial to illustrate the non-inferiority of PCSK9 inhibitor efficacy for preventing ANT-induced cardiotoxicity when compared to the current medical standard DZR.

Statins have been investigated in clinical trials due to their pleiotropic effects, similar to PCSK9 inhibitors, with conflicting results. Two clinical trials, namely the Preventing Anthracycline Cardiovascular Toxicity with Statins (PREVENT) trial and the Statins for the Primary Prevention of Heart Failure in Patients Receiving Anthracycline Pilot Study (SPARE-HF), administered 40 mg of atorvastatin to patients with various malignancies and undergoing anthracycline-based treatments. Both trials evaluated LVEF using cardiac magnetic resonance (cMR) imaging at 24 months and within 4 weeks of anthracycline completion, respectively, to assess significant changes from the baseline between treatment groups. However, neither found any significant difference. On the other hand, the Statins to Prevent the Cardiotoxicity of Anthracyclines (STOP-CA) trial found a significantly lower incidence of anthracycline-induced cardiotoxicity, as measured by cMR-measured LVEF, in statin groups compared to the placebo group. Interestingly, despite administering a higher median dose of anthracyclines (300 mg/m^2^) compared to the PREVENT and SPARE-HF trials (240 mg/m^2^), the STOP-CA trial showed cardioprotection. This finding is not consistent with the expected dose-dependent cardiotoxicity of anthracyclines. These divergent results may be explained by factors such as underpowered studies, high dropout rates, the inclusion of other neurohormonal antagonists, and variations in statin treatment duration. This suggests that the non-LDL-based mechanisms of statins, as well as PCSK9 antagonism, may possess important cardioprotective properties that warrant further investigation. Based on the findings of the STOP-CA trial and clinical perspectives, it is reasonable to consider initiating statins in higher-risk patients [31].

Other pharmacologic agents have shown promise with regard to preventing ANT-induced cardiotoxicity, in addition to PCSK9 inhibitors. Cyclosporine A has been shown in preclinical studies to decrease the influx of Ca^2+^ into the mitochondria via inhibition of mPTP formation. A murine study [70] investigating acute and sub-chronic DOX exposure revealed decreased mitochondrial fragmentation and improved adenosine triphosphate production with treatment of cyclosporine A. Additional studies have shown that targeting other parts of oxidative phosphorylation (cytochrome C oxidase, cardiolipin, Sirtuin-1), may have benefits for reducing mitochondrial damage induced by DOX [71]. Though the likelihood of safety with cyclosporine A is high, its therapeutic potential has not been thoroughly explored in human trials. A reasonable next step in the evaluation of cyclosporine A’s efficacy for the prevention of cardiovascular toxicity secondary to doxorubicin would be to perform a small time-limited trial with serologic markers to assess generalized inflammation and cardiac-specific markers. It is also possible that PCSK9 inhibitors and cyclosporine A may one day be used in conjunction to synergistically provide optimal cardiovascular protection in the setting of ANT-induced cardiotoxicity.

Interestingly, PCSK9 inhibitors have also been postulated to provide cardioprotection from immune-related atherosclerotic vascular events secondary to immune checkpoint inhibitors (ICIs) [72]. ICIs are associated with a more than threefold increase in atherosclerotic cardiovascular events and accelerated progression of aortic plaques, likely due to heightened systemic inflammation [73]. Drobnis et al. showed that patients receiving statins along with ICI treatment experienced a ~50% reduction in plaque progression compared to those receiving ICI therapy alone [73]. PCSK9 inhibitors stabilize plaques and induce significant regression to a greater extent than statins [74,75]. Corroborating their known pleiotropic effects, preclinical studies have demonstrated that PCSK9 inhibition synergistically enhances immunotherapy’s anti-tumor effects through LDL-receptor-independent pathways [76,77]. These medications not only have the potential to provide cardioprotection through immunotherapy-based regimens against cardiovascular events, but also to enhance treatment response rates.

In the present era of cardiovascular medicine where high-quality evidence is used to justify patient care guidelines, it is sobering to realize that only 3% of cardio-oncology Class I recommendations are supported by Level A evidence [78]. This suggests that the majority of cardio-oncology patient management decisions are not founded on literature-supported evidence, though this is likely confounded by the highly personalized nature of oncologic treatment plans. This low percentage of Level A evidence of Class I cardio-oncology recommendations is contrasted with 8.5% for the general American College of Cardiology/American Heart Association guidelines and 14.3% for European Society of Cardiology guidelines [79]. As researchers and clinicians strive to enhance evidence-based clinical reasoning in the evolving field of cardio-oncology and improve cancer patient care, the evidence presented in this review may inspire further exploration of pharmaceuticals that can mitigate ANT-induced cardiotoxicity.

## 5. Conclusions

Anthracyclines such as doxorubicin, though useful in the management of oncologic phenomena, have well-known cardiovascular complications. Heightened inflammation, reactive oxygen species, nucleic acid damage, and cellular respiration dysfunction are the primary vehicles that lead to cardiac dysfunction and accelerated vascular disease. PCSK9 inhibitors work on similar molecular targets, and exhibit strong potential to serve as useful prophylactic cardioprotective agents for patients undergoing chemotherapy regimens that include anthracyclines. Additional clinical investigation is needed to elucidate the full potential of PCSK9 inhibitors or other similar pharmacologic agents.

## Figures and Tables

**Table 1.** Antioxidants Evaluated for Cardioprotection from Anthracycline-induced Toxicity. Four major antioxidants are highlighted with the relevant associated references. The mechanism of action is described as well as the hypothesized cardioprotective effect. Finally, the result is described; none of the four antioxidants had clinically significant effects with regard to ANT-induced cardiotoxicity.

Compound [Reference #]	Basic Mechanism of Action	Speculated Cardioprotective Effect	Result
*N*-acetylcysteine [37,38]	Precursor of L-cysteine, stimulates intracellular glutathione synthesis, and prevents activation of pro-inflammatory cytokines	Increases glutathione production, enhances antioxidant activity, and mitigates myocyte damage	*N*-acetylcysteine did not reverse ANT-induced cardiotoxicity in disease-free sarcoma patients and showed no change in heart failure incidence
Glutathione [39]	Reduces cellular damage and oxidative stress by directly interacting with free radicals and intracellular processes via ROS scavenging, regenerating antioxidants, and acting as a redox buffer to maintain cellular balance	Decreases the amount of oxidative stress/free radical formation triggered by acute and chronic cardiac disease processes such as myocardial infarction, ischemia/reperfusion injury, toxin-mediated damage, and heart failure	Oral glutathione did not protect against local or systemic oxidative stress induced by ANT in women with breast cancer
Vitamin E [40]	Interrupts the destruction mediated by free radicals as well as lipid oxidation	Decreased oxidation of LDL in coronary vasculature	Vitamin E is ineffective in preventing ANT-induced cardiotoxicity
Flavonoids [41]	Blocks certain intracellular ROS-generating enzymes and reduces inflammation via inhibition of prostaglandin synthesis and iNOS	Reduction of oxidative stress during cardiomyocyte injury	High-dose flavonoids potentially worsen ANT-induced cardiotoxicity

Abbreviations: ANT = anthracycline; LDL = low-density lipoprotein; ROS = reactive oxygen species; iNOS = inducible nitric oxide synthase.

## Abbreviations

ANT: Anthracycline
Ca^2+^: Calcium
DNA: Deoxyribonucleic acid
DOX: Doxorubicin
ETC: Electron Transport Chain
EMPACT: Empagliflozin in the Prevention of Cardiotoxicity in Cancer Patients Undergoing Chemotherapy Based on Anthracyclines
ECM: Extracellular matrix
ICIs: Immune checkpoint inhibitors
IL: Interleukin
I/R: Ischemia-reperfusion
LOX-1: Lectin-like oxidized low-density lipoprotein receptor-1
LDL: Low-density lipoprotein
mg/m^2^: Milligrams per square meter
mitDNA: Mitochondrial DNA
mPTP: Mitochondrial Permeability Transition Pore
MyD88: Myeloid Differentiation primary response gene 88
NADPH: Nicotinamide adenine dinucleotide phosphate
NOD: Nucleotide oligomerization domain
NLRP3: NOD-like Receptor Protein Inflammasome
NF-κB: Nuclear Factor kappa-light-chain-enhancer of activated B cells
Ox: Oxidized
PCSK9: Proprotein convertase subtilisin/kexin type 9
ROS: Reactive oxygen species
SGLT2is: Sodium-Glucose Cotransporter-2 inhibitors
CANTOS: The Canakinumab Antiinflammatory Thrombosis Outcome Study
TLRs: Toll-like receptors
Top2: Topoisomerase II
